# Optical properties and Judd–Ofelt analysis of Dy^3+^ doped CoAl_2_O_4_ nanocrystals

**DOI:** 10.1039/d4na00537f

**Published:** 2024-08-26

**Authors:** N. T. Hien, N. T. Kien, V. H. Yen, T. Ngoc, P. V. Do, V. X. Phuc, N. X. Ca

**Affiliations:** a Institute of Science and Technology, TNU – University of Sciences Thai Nguyen Vietnam nguyenxuanca@tnus.edu.vn; b Faculty of Engineering and Technology, TNU – University of Information and Communication Technology Thai Nguyen Vietnam; c Institute of Research and Development, Duy Tan University Da Nang 550000 Vietnam; d Faculty of Environmental and Natural Sciences, Duy Tan University Da Nang 550000 Vietnam; e Thuyloi University 175 Tay Son, Dong Da Hanoi Vietnam; f People's Council of Kien An Hai Phong Vietnam

## Abstract

CoAl_2_O_4_:*x*Dy^3+^ nanocrystals (*x* = 0, 0.1, 0.5, 1.0, and 3.0 mol%) with spinel structures were prepared using the co-precipitation method. The crystal structure, optical properties, and presence of elements were respectively analyzed using X-ray diffraction, photoluminescence excitation, photoluminescence spectra, luminescence lifetime, and X-ray photoelectron spectroscopy. The influence of temperature on material fabrication was studied using differential scanning calorimetry and thermogravimetric techniques. The color characteristics of Dy^3+^ luminescence in the CoAl_2_O_4_ host were evaluated using CIE chromaticity coordinates and correlated color temperature. For the first time, the electronic dipole transitions in the photoluminescence excitation spectra were used to calculate the optical parameters of Dy^3+^ ions in the CoAl_2_O_4_ host using Judd–Ofelt theory. The Inokuti–Hirayama model was used to explain the energy transfer process between Dy^3+^ ions, the main interaction mechanism, and energy transfer parameters for the luminescence of Dy^3+^ ions.

## Abstract 2

The energy level diagram and cross-relaxation channels of Dy^3+^ ions in CoAl_2_O_4_ NCs.

## Graphical abstract

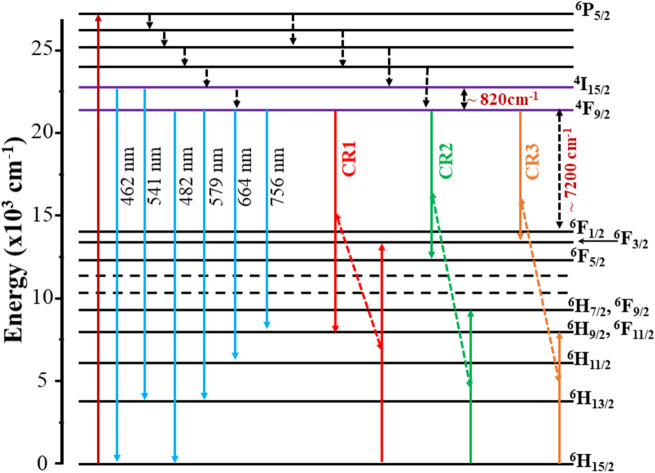

## 1 Introduction

Spinel oxides with the general chemical formula AB_2_O_4_ include several materials. Their outstanding feature is their special crystal structure. The structure is named after the mineral spinel and involves a specific arrangement of metal cations (A and B) and oxygen anions. These materials have been widely used in ceramic pigments, magnetic materials, printing technologies, and catalysts.^1–3^ Cobalt aluminate (CoAl_2_O_4_), commonly known as Thenard blue, is a spinel-type oxide known for its intense blue color and various applications. It is known for its bright blue color, which results from the electronic transitions of Co^2+^ ions in the tetrahedral crystal field.^4^ In the visible light region, the optical properties of CoAl_2_O_4_ are primarily influenced by the d–d transitions of Co^2+^ (3d^7^) electrons in the tetrahedral (Td) structure. Hybridization between the O-2p and Co-3d orbitals permits dipole-forbidden d–d transitions.^5^ The optical, electromagnetic, and color properties of the CoAl_2_O_4_ material can be effectively changed by adjusting the p–d hybridization. The simplest way to change the p–d hybridization in the CoAl_2_O_4_ compound is to dope it with a transition metal or rare earth ions. Doping transition metals and rare earth ions into the CoAl_2_O_4_ host can change its color and optical and magnetic properties.^3,5^

Pradhan *et al.* fabricated and studied the spectroscopic and magnetic properties of spin-frustrated Mn-doped CoAl_2_O_4_ spinel.^5^ Research shows that the absorption spectra associated with d–d transitions are altered by Mn doping at the Co site. Random substitution of Mn introduces various magnetic exchange pathways of differing magnitudes, which enhance the interactions between the magnetic ions. Competitive magnetic exchange interactions between Co^2+^ and Mn^2+^ ions give rise to the magnetic properties of the material. Wang and colleagues suggested that the luminescence properties of CoAl_2_O_4_ are enhanced when doped with Ce and Mn ions.^6^ The blue color diminishes as the number of doped ions increases. The photoluminescence enhancement in Ce and Mn co-doped CoAl_2_O_4_ is attributed not to the energy transfer between Ce^4+^ and Mn^4+^, but to surface or impurity defects. Tong *et al.* suggested that when the doping amount is less than 10%, Al^3+^ can be partially replaced by trivalent rare earth ions (RE^3+^), forming spinel CoAl_2−*x*_RE_*x*_O_4_ complex oxides.^7^ Replacing Al^3+^ with RE^3+^ in CoAl_2_O_4_ enhances the blueness of the pigments. Er^3+^ ions can enhance the upconversion luminescence intensity of the CoAl_2_O_4_ pigment. The luminescence appeared blue, and variations in the luminescence intensity were observed with different Er doping concentrations.^8^ The degradation of colored and colorless organic dyes under natural sunlight was investigated using CoAl_2_O_4_ and Ni-doped CoAl_2_O_4_. The Ni-doped CoAl_2_O_4_ demonstrated higher photocatalytic activity compared to the undoped CoAl_2_O_4_, achieving photodegradation rates of 92.7% for crystal violet, 88.77% for methylene blue, and 63.34% for benzoic acid.^9^

Trivalent dysprosium (Dy^3+^) is a rare earth ion that has many important applications in optical devices and materials. Its applications include lighting, telecommunications, spectroscopy, and solar energy, making it a crucial element in the development of next-generation optical devices and materials.^10,11^ Dy^3+^ ions are capable of generating white light because of their unique luminescence properties. This was achieved through a combination of intense blue (484 nm, ^4^F_9/2_ → ^6^H_15/2_) and yellow (575 nm, ^4^F_9/2_ → ^6^H_13/2_) emissions when doped into various crystals, glasses, and glass-ceramic materials.^12,13^ Dy^3+^-doped solid-state systems can be efficiently excited by commercial ultraviolet (UV) or blue light-emitting diodes (LEDs), because there is a spectral overlap between the excitation spectra of Dy^3+^ ions and the emission spectra of these LEDs.^13^ This makes them practical for use in modern lighting technology. Dy^3+^ ions have been extensively studied in crystals, glasses, and glass-ceramic materials owing to their well-established optical properties and simple energy structures.^13,14^ These materials serve as effective hosts for Dy^3+^ ions to exhibit luminescence properties. Beyond their practical applications, Dy^3+^ ions are used as spectroscopic probes to study the local symmetry and bonding features of other RE^3+^ ions within different host matrices.^15^ The yellow-to-blue emission ratio of Dy^3+^ can vary depending on factors such as the nature of the host material and the concentration of Dy^3+^ ions. Dy^3+^-doped phosphors have been used for light-emitting diodes, especially white light-emitting diodes by adjusting the yellow-to-blue ratio of Dy^3+^ ions. Dy^3+^ ions stand out not only for their ability to generate white light but also for their role in advancing optical technologies through their use in various luminescent materials.^16,17^

Judd–Ofelt (JO) theory is a powerful tool for analyzing the local environment around RE^3+^ ions and predicting their optical properties. Traditional JO analysis relies on absorption spectra to determine the Judd–Ofelt parameters (*Ω*_*λ*_). However, this approach can be challenging for powder materials. To solve this problem, recent studies have proposed alternative methods for calculating the *Ω*_*λ*_ parameters, using different spectral types such as diffuse-reflection spectrum, fluorescence decay curve, and photoluminescence excitation (PLE) spectrum. In the above spectra, the PLE spectrum is particularly versatile as it can be obtained for various material morphologies, including films, nanocrystalline structures, bulk crystals, and glasses. In this study, the PLE spectra of Dy^3+^ ions were utilized to calculate the *Ω*_*λ*_ parameters of CoAl_2_O_4_:*x*Dy^3+^ NCs. The advantage of using PLE spectra lies in their applicability to a wide range of sample types, circumventing the challenges associated with traditional absorption-based JO analysis. This method provides a more accessible route to determine the *Ω*_*λ*_ parameters, offering valuable insights into the optical behavior and local structure of the Dy^3+^ ions in the CoAl_2_O_4_ host. A series of CoAl_2_O_4_:*x*Dy^3+^ nanocrystals (NCs) (*x* = 0, 0.1, 0.5, 1.0, and 3.0 mol%) were prepared using the co-precipitation method. For the first time, the electronic dipole transitions in the PLE spectra were used to calculate the optical parameters of Dy^3+^ ions in the CoAl_2_O_4_ host using JO theory. The Inokuti–Hirayama model was used to explain the energy transfer process between Dy^3+^ ions and to determine the main interaction mechanism as well as the energy transfer parameters for the luminescence of Dy^3+^ ions.

## 2 Experimental

### 2.1. Synthesis of CoAl_2_O_4_:Dy^3+^ nanocrystals

**Fig. 1:**
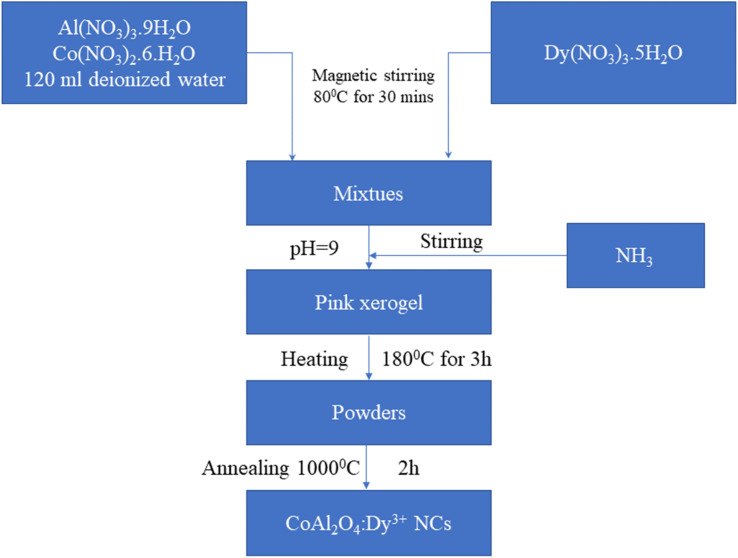
Schematic flow chart for the synthesis of CoAl_2_O_4_:Dy^3+^ NCs.

The synthesis process of the CoAl_2_O_4_:Dy^3+^ NCs *via* the co-precipitation method is shown in Fig. 1. First, 0.05 mol Co(NO_3_)_2_·6H_2_O and 0.1 mol Al(NO_3_)_3_·9H_2_O were dissolved in 120 ml deionized water under magnetic stirring at 80 °C for 30 minutes and then, Dy(NO_3_)_3_·5H_2_O was added at molar ratios of 0.1, 0.5%, 1%, and 3% of Co(NO_3_)_2_·6H_2_O. Next, an NH_3_ solution was added to the previous solution to adjust the pH to approximately 9. The mixture was then stirred at 80 °C until a pink xerogel formed. The xerogel was subsequently heat-treated at 180 °C for 3 hours to eliminate a significant amount of organic solvent and water. Finally, the dried xerogel was ground to a fine powder and calcined in air at 1000 °C for 2 hours. The CoAl_2_O_4_, CoAl_2_O_4_:0.1% Dy^3+^, CoAl_2_O_4_:0.5% Dy^3+^, CoAl_2_O_4_:1% Dy^3+^, and CoAl_2_O_4_:3% Dy^3+^ samples were denoted as S0, S0.1, S0.5, S1, and S3, respectively.

### 2.2. Characterization

The crystal structures of the synthesized samples were analyzed using X-ray diffraction (XRD) (D5005) over a 2*θ* range of 20° to 80°, with a scan speed of 2° min^−1^ and equipped with a Cu-Kα radiation source. The crystal lattice structure of the CoAl_2_O_4_:Dy^3+^ NCs was drawn using Vesta software. X-ray photoelectron spectroscopy (XPS) was performed using a Thermo VG Escalab 250 photoelectron spectrometer. The PLE spectra, photoluminescence (PL) spectra, and luminescence lifetimes were recorded using an FLS1000 spectrophotometric system that covers a range of 230 nm to >1000 nm with a 450 W Xe lamp and pulsed diode lasers.

## 3 Results and discussion

### 3.1. DSC and TG thermoanalytical techniques

**Fig. 2:**
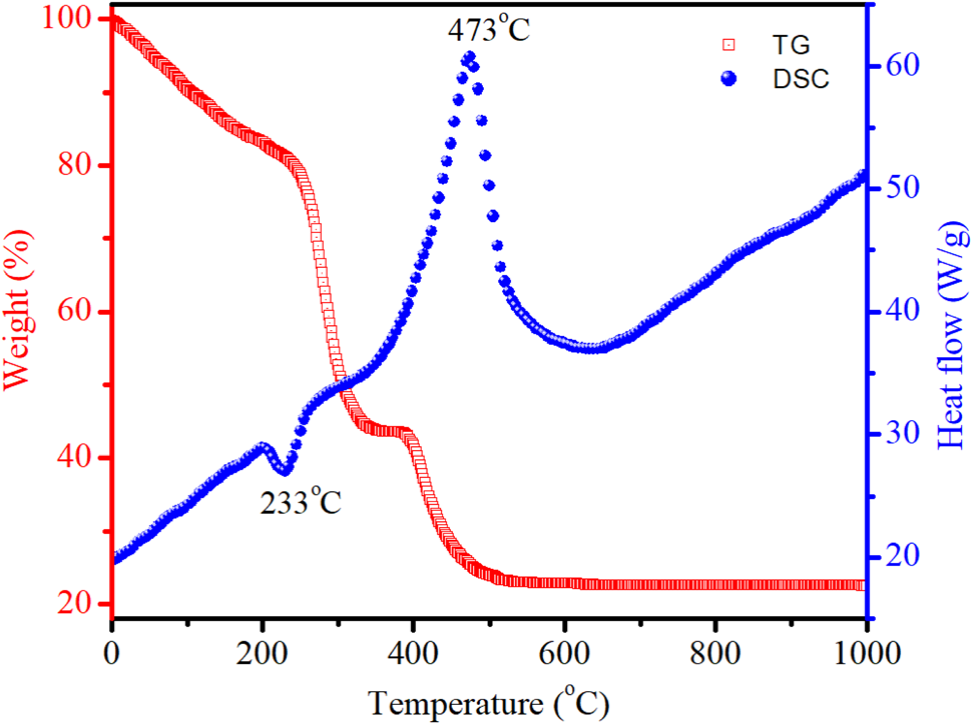
DSC/TG curves of the CoAl_2_O_4_ precursor gel.

Differential Scanning Calorimetry (DSC) is a thermoanalytical technique used to study the thermal behavior of materials. DSC measures the amount of heat required to increase the temperature of a sample compared to a reference. This technique can provide insights into thermal transitions such as melting, crystallization, glass transitions, and other thermal events. Thermogravimetric (TG) analysis is another important technique used to study the thermal stability and composition of materials by measuring changes in mass as a function of temperature or time. This technique investigates processes such as dehydration, decomposition, and oxidation. Fig. 2 displays the TG/DSC curves of the dried gel. The endothermic peak at 233 °C results from the decomposition of nitrates, leading to substantial weight loss. The exothermic peak at 473 °C indicated the crystallization of spinel CoAl_2_O_4_. When the temperature exceeded 520 °C, no significant weight loss was observed.

### 3.2. X-ray diffraction studies

**Fig. 3:**
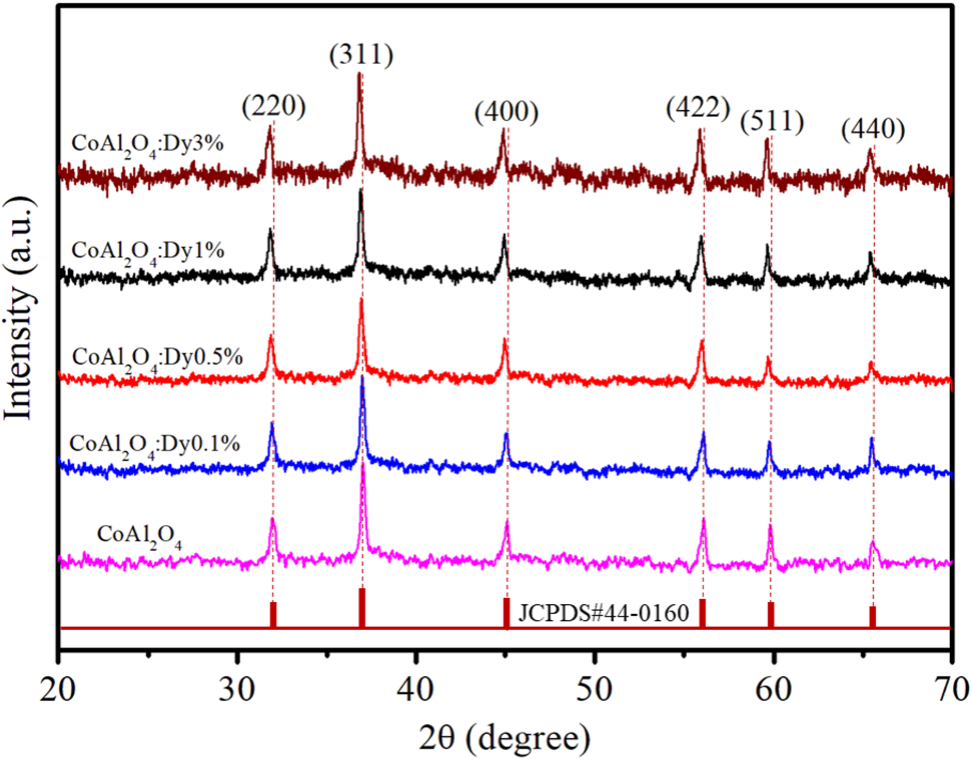
XRD patterns of pure CoAl_2_O_4_ and CoAl_2_O_4_:Dy^3+^ NCs.

**Fig. 4:**
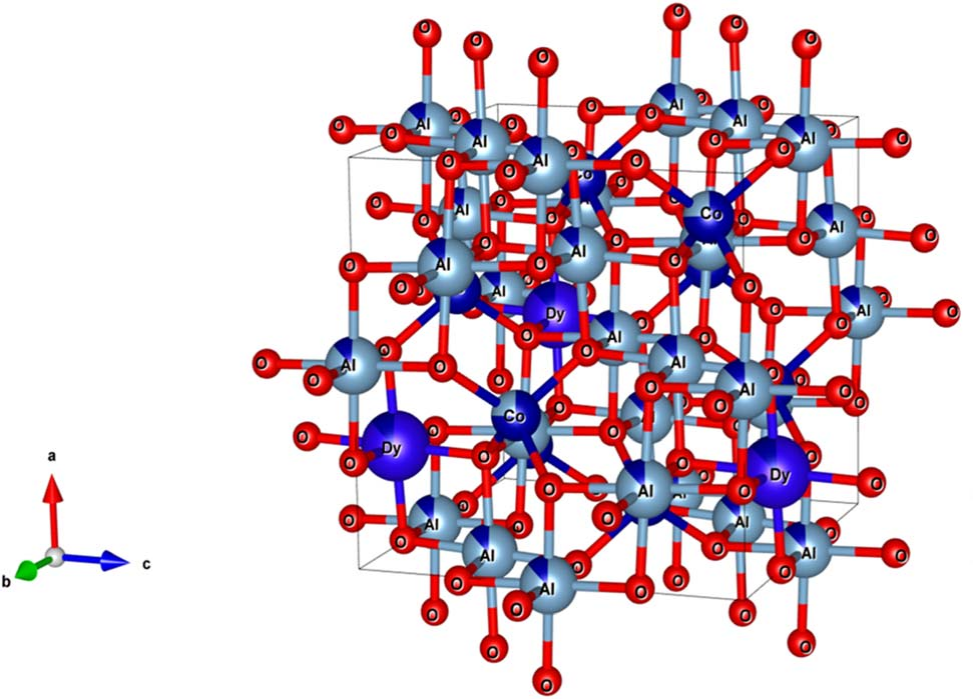
The unit-cell scheme of CoAl_2_O_4_:Dy^3+^ NCs (spinel structure).

The XRD analysis of the samples provides comprehensive information about their crystalline structure, phase purity, crystallite size, and lattice parameters, which are critical for understanding their physical and chemical properties. The XRD patterns of the samples are shown in Fig. 3. With pure CoAl_2_O_4_ NCs, the diffraction peaks observed at 2*θ* angles of 31.91, 36.83, 45.11, 56.32, 59.93 and 65.91° correspond to the lattice planes (220), (311), (400), (422), (511), and (440) (JCPDS card no. 44-0160, spinel structure, and space group *Fd*3̄*mz*).^1,3,5^ The structure of the CoAl_2_O_4_ host did not change when doped with Dy^3+^ ions and no other diffraction peaks of Dy oxide were observed. XRD showed the formation of a spinel phase with a 2*θ* angle of 36.83° corresponding to *d* = 0.244 nm.^18^ The XRD observation results show that the diffraction peaks slightly shifted to a smaller angle as the Dy doping content increased. The lattice parameters of the samples were calculated and are listed in Table 1. As the Dy doping concentration increases, the lattice parameters of CoAl_2_O_4_ increase demonstrating that Dy doping causes lattice expansion. Co^2+^, Al^3+^ and Dy^3+^ ions have radii of 0.054, 0.0675, and 0.091 nm, respectively.^19,20^ The lattice constants of CoAl_2_O_4_ increase with increasing Dy concentration because the ionic radius of Dy^3+^ is significantly larger than those of Co^2+^ and Al^3+^. This result indicates that all Dy^3+^ ions were successfully incorporated into the CoAl_2_O_4_ host lattice. According to the principle that elements will preferentially replace other elements with similar valences, Dy replaces Al to create the structure CoAl_2−*x*_Dy_*x*_O_4_ (see Fig. 4). According to Pauling's rule, the coordination number of Dy is 6, forming an octahedral structure (DyO_6_).^8^ Tong *et al.* also suggested that Al^3+^ can be partly replaced by Re^3+^ when the doping amount is less than 10%, which forms spinel CoAl_2−*x*_RE_*x*_O_4_ complex oxides.^7^

**Table 1:** The diffraction angle (2*θ*), lattice constants (*a*), cell volume (*V*), *β*, crystallite size (*D*), and crystallite strain (*ε*) of NCs

Sample	2*θ* (311)	*β* × 10^−2^ (rad)	*a* (nm)	*V* (nm^3^)	*D* (nm)	*ε* × 10^−3^
S0	36.829	0.632	0.801	0.514	24.183	1.432
S01	36.803	0.641	0.820	0.551	23.991	1.444
S05	36.735	0.638	0.826	0.563	24.518	1.413
S1	36.649	0.645	0.843	0.599	24.833	1.395
S3	36.563	0.649	0.865	0.647	25.335	1.368

The broadening of the diffraction peaks can be analyzed using the Scherrer equation to estimate the crystallite size:^18–20^1
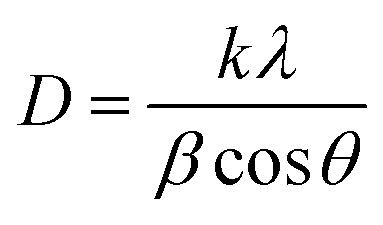
where *D* is the average crystallite size, *k* is the shape factor (usually around 0.9), *λ* is the wavelength of the X-ray (∼0.154 nm), *β* is the full width at half maximum (FWHM) of the peak, and *θ* is the Bragg angle. The positions of the peaks were used to calculate the lattice parameters of the crystal structure. For a spinel structure, such as CoAl_2_O_4_, the lattice parameter *a* can be derived from the peak positions using the following relationship for a cubic crystal system:^18,19^2
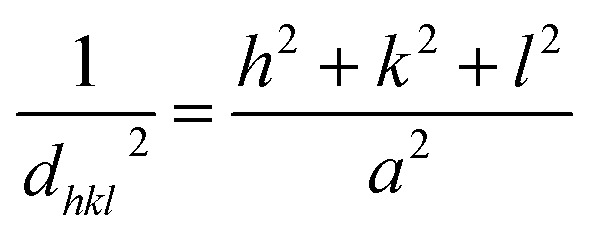
where *d*_*hkl*_ is the interplanar spacing, and *h*, *k*, and *l* are the Miller indices of the planes. *d*_*hkl*_ was calculated using Bragg's equation:^19,20^

3 *nλ* = 2*d*_*hkl*_ sin *θ*

The unit cell volume of the NCs with cubic structures was calculated using the following equation:^19^

4 *V* = *a*^3^

Peak broadening analysis can also provide information about microstrains and defects within the crystal lattice. The effective crystallite strain in the NCs was determined using the Stokes–Wilson equation.^19^5
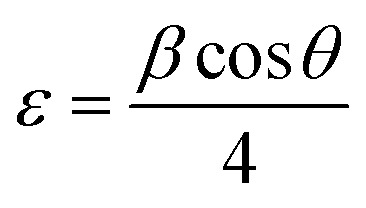


The calculated crystal lattice parameters of the samples are listed in Table 1. The crystallite sizes of the CoAl_2_O_4_ and CoAl_2_O_4_:Dy^3+^ NCs did not change significantly, with values ranging from 23 to 25 nm.

### 3.3. Elemental and chemical composition analysis

**Fig. 5:**
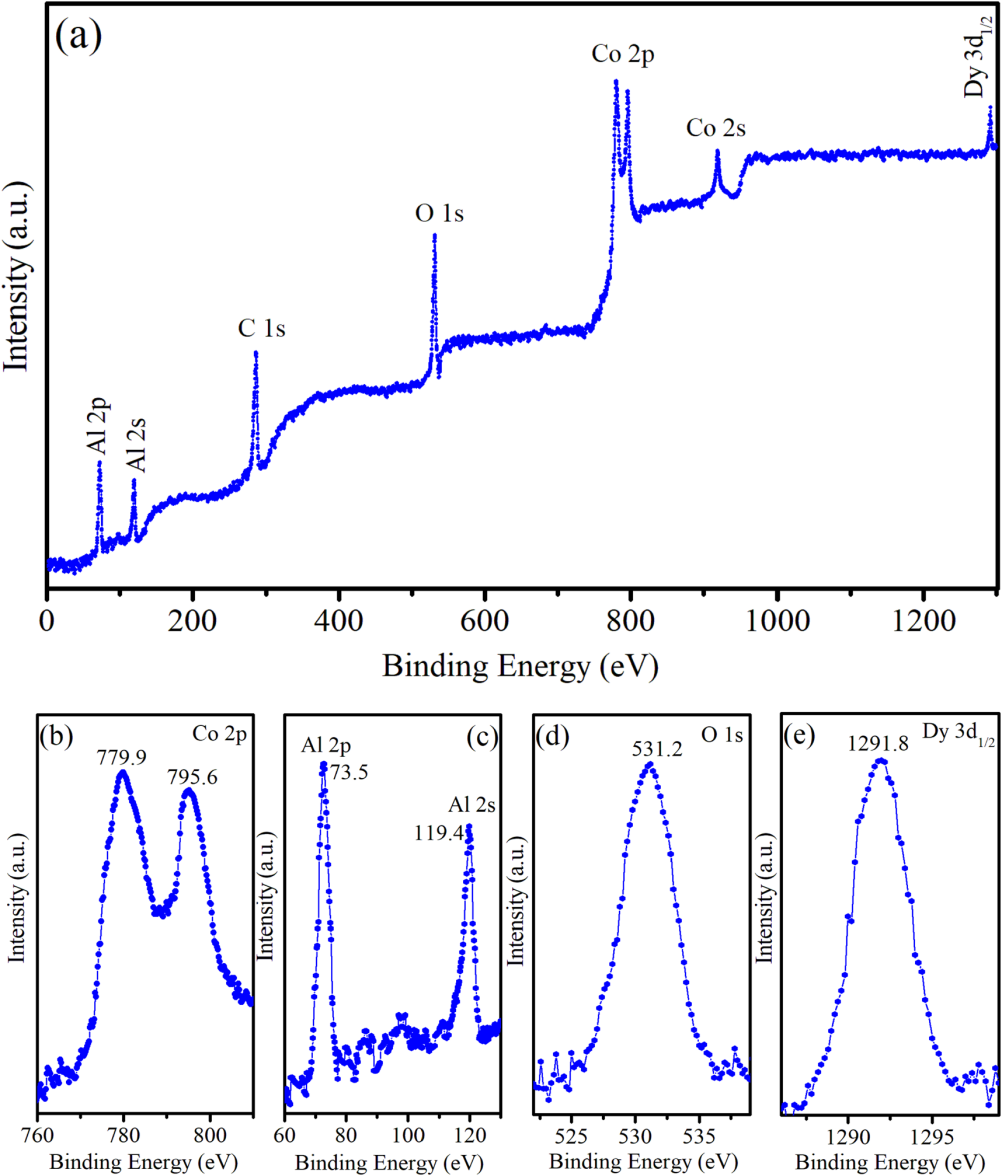
(a) Survey XPS spectrum of CoAl_2_O_4_:Dy3% NCs, (b) Co 2p, (c) Al 2p, (d) O 1s, and (e) Dy 3d.

The XPS survey spectrum provides comprehensive information on the elemental composition of the sample, including the chemical states of the elements present. For CoAl_2_O_4_:Dy3% NC, the XPS survey spectrum showed characteristic peaks corresponding to the elements Co, Al, O, Dy, and C (see Fig. 5). The presence of C in the sample was attributed to the organic precursors used in the sample preparation process. Fig. 5b presents the high-resolution XPS spectrum of Co 2p, which has two peaks at 779.9 eV and 795.6 eV, corresponding to Co 2p_3/2_ and Co 2p_1/2_, respectively. The positions of the energy peaks and the energy difference of 15.7 eV between these peaks are typical of divalent Co ions. The relatively narrow and symmetric Co 2p spectrum demonstrated that Co^2+^ ions occupied octahedral positions in the synthesized samples.^19^Fig. 5c presents the high-resolution XPS spectra of Al 2p (at 73.5 eV) and Al 2s (119.4 eV). The Al 2p value was similar to those reported by Duan^21^ (73.26 eV) and Peng^22^ (74 eV). This Al 2p value confirms that Al is trivalent in the CoAl_2_O_4_ NCs. The O 1s peak was asymmetric and fixed at 531.2 eV (Fig. 5d), which aligns with the O 1s peak in CoAl_2_O_4_ NCs reported by Peng *et al.*^22^ The XPS spectrum of the Dy^3+^ ion typically showed characteristic peaks corresponding to the 3d and 4d orbitals. The Dy 3d region often exhibits three prominent peaks, corresponding to the 3d_5/2_, 3d_3/2_, and 3d_1/2_ spin–orbit components. These peaks were observed because of the spin–orbit splitting of the Dy 3d core level. The Dy 3d_5/2_ and 3d_3/2_ peaks are outside the measurement range (greater than 1300 eV). The position of the Dy 3d_1/2_ peak (in Fig. 5e) is at 1291.8 eV and its shape shows asymmetry. This indicates Dy in the trivalent state in the Dy-doped CoAl_2_O_4_ NCs and complex interactions with the surrounding host.^19,21^

### 3.4. Judd–Ofelt analysis of CoAl_2_O_4_:Dy^3+^ NCs

**Fig. 6:**
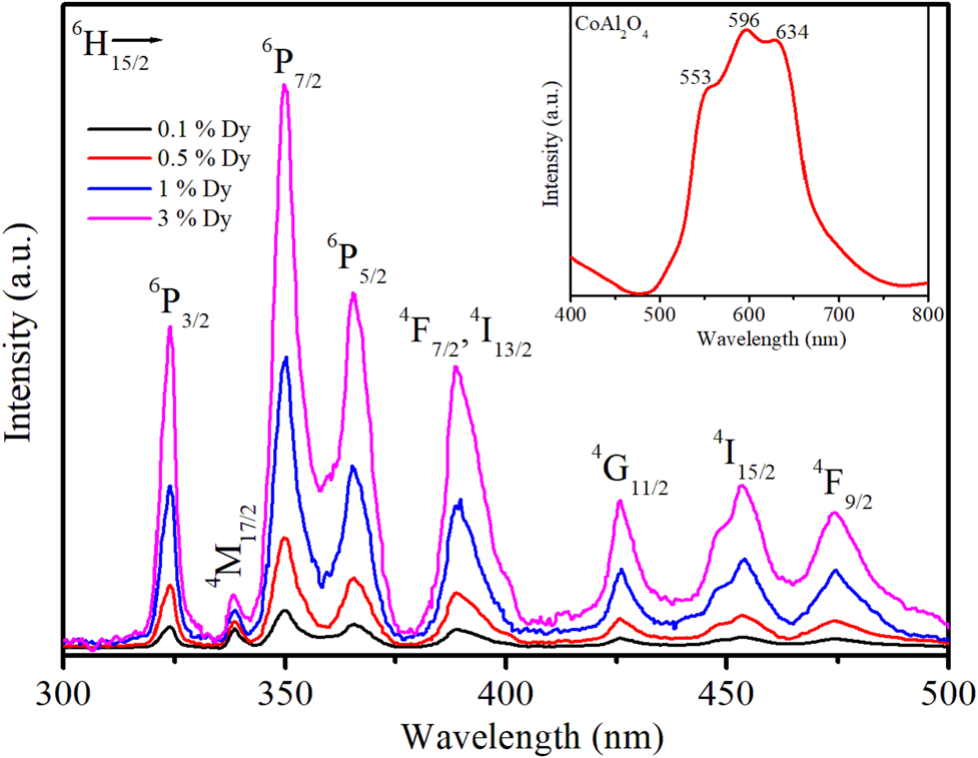
Illustration of the PLE spectra of the Dy^3+^ ion in CoAl_2_O_4_:Dy^3+^ NCs; the emission signal was monitored at a wavelength of 579 nm (^4^F_9/2_ → ^6^H_13/2_ transition).

The PLE spectra of the samples are shown in Fig. 6. To obtain these spectra, the luminescence signal was fixed at an emission wavelength of 579 nm, whereas the excitation wavelength was varied from 300 to 500 nm. Cobalt aluminate's color and PLE are primarily due to electronic transitions within the Co^2+^ ions. The electronic structure and crystal field effects influence the PLE characteristics. Co^2+^ in the tetrahedral sites of the spinel structure undergoes d–d electronic transitions, which are typically in the visible range, contributing to its blue color. Splitting of the d-orbitals in a tetrahedral field results in specific absorption bands that correspond to the energy difference between the split d-orbitals. The PLE spectrum of sample S_0_ exhibited three peaks at 549, 594, and 637 nm (inset in Fig. 6). According to the literature, these three peaks are attributed to ^4^A_2_(F) → ^4^T_1_ (P) transitions that arise from the Jahn–Teller distortion of the Td structure^23^ and the presence of Co^2+^ ions arranged in a ligand field with a 3d^7^ electron configuration.^24^ The typical PLE peak at 594 nm (yellow-orange region) gives the material a distinctive blue appearance by absorbing light in the yellow-orange spectrum.

As shown in Fig. 6, the PLE spectra of Dy^3+^ in the CoAl_2_O_4_:Dy^3+^ NCs exhibited eight excitation peaks at 324, 337, 350, 363, 387, 426, 451, and 473 nm. These PLE bands correspond to the intra-configuration 4f^9^ transitions of the Dy^3+^ ion from the ^6^H_15/2_ ground state to the excited states of ^6^P_3/2_, ^4^M_17/2_, ^6^P_7/2_, ^6^P_5/2_, ^4^F_7/2_+^4^I_13/2_, ^4^G_11/2_, ^4^I_15/2_, and ^4^F_9/2_, respectively.^8,19^ Notably, the PLE bands at 350 and 363 nm exhibited higher intensities than the others, making them the preferred choice for exciting the luminescence of the Dy^3+^ ion.

#### 3.4.1. Determination of the intensity parameters

JO is an effective theory for studying the structure of the local medium around RE^3+^ ions and predicting their optical properties.^19^ In this theory, the intensity parameters *Ω*_*λ*_ (*λ* = 2, 4, 6) are considered the key to calculating other important optical parameters. Traditional JO analysis typically utilizes the absorption spectrum to calculate the *Ω*_*λ*_ parameters. Thus, this method is especially suitable for the JO analysis of bulk samples, such as single crystals and glasses. However, the traditional calculation route is very difficult to apply to powder materials because of the difficulty in quantitatively recording the absorption spectra and determining the optical path length for these samples. To overcome this obstacle, some authors have recently proposed new methods for computing the *Ω*_*λ*_ parameters. According to these methods, the *Ω*_*λ*_ parameters can be calculated by utilizing the diffuse-reflection spectrum,^25^ fluorescence decay curve,^26^ and PLE spectrum.^27^

In this study, the electronic dipole transitions in the PLE spectra of Dy^3+^ ions were used to calculate the *Ω*_*λ*_ parameters of CoAl_2_O_4_:Dy^3+^ NCs. Because the PLE spectra can be easily recorded for any sample (*e.g.* films, nanocrystals, bulk crystals, and glasses), the calculation of the *Ω*_*λ*_ parameters can avoid the difficulty arising from the material morphology. For this route, the experimental line strength (*S*_exp_) of the transition from the J state to the J′ state is calculated from the PLE spectrum using the following formula:^28^6
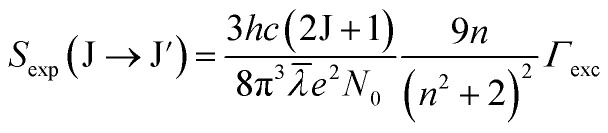
where *c* is the speed of light in vacuum, *h* is Planck's constant, *n* is the refractive index of the material (for the CoAl_2_O_4_ host, *n* is approximately 1.4, which is considered constant across all wavelengths^29^), *λ* is the mean wavelength of the PLE band, *N*_0_ is the concentration of Dy^3+^ ions, and *Γ*_exc_ is the integrated excitation intensity of the excitation band.

On the other hand, the line strength of an electric dipole (ED) transition is computed using JO theory:^30^7
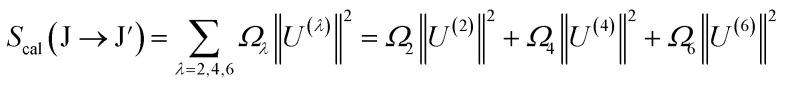
where *Ω*_2,4,6_ are the JO intensity parameters, and the ‖*U*^(*λ*)^‖^2^ symbols are the doubly reduced matrix elements of the unit tensor with a rank of *λ* = 2, 4, 6 for the J → J′ transitions. These parameters are almost independent of the host matrices and have been described in previous publications.^31^

From the PLE spectra of Dy^3+^ in CoAl_2_O_4_:Dy^3+^, the *Γ*_exc_ values were calculated for all bands, and the experimental line strengths of these transitions were calculated using eqn (6) and (7). The JO intensity parameters *Ω*_*λ*_ (*λ* = 2, 4, 6) are determined by solving the system of equations *S*_cal_ = *S*_exp_ using the least squares method. The obtained results for the CoAl_2_O_4_:Dy^3+^ NCs are listed in Table 2 and are compared to those of Dy^3+^ ions doped into some crystal lattices. The errors in the fitting procedures for the *Ω*_*λ*_ parameters of all samples were lower than 12%. This value is within the intrinsic error region (approximately 20%) of JO theory.

**Table 2:** The JO intensity parameters (*Ω*_*λ*_, *λ* = 2, 4, 6) of Dy^3+^ ions in some hosts

Samples	*Ω*_2_ (10^−20^ m^2^)	*Ω*_4_ (10^−20^ m^2^)	*Ω*_6_ (10^−20^ m^2^)	Trend	References
S01	4.72 ± 0.56	2.69 ± 0.32	3.67 ± 0.43	*Ω*_2_ > *Ω*_6_ > *Ω*_4_	This work
S05	4.86 ± 0.39	2.65 ± 0.28	3.89 ± 0.32	*Ω*_2_ > *Ω*_6_ > *Ω*_4_	This work
S1	5.25 ± 0.41	3.12 ± 0.27	4.15 ± 0.34	*Ω*_2_ > *Ω*_6_ > *Ω*_4_	This work
S3	4.73 ± 0.45	3.05 ± 0.31	3.98 ± 0.44	*Ω*_2_ > *Ω*_6_ > *Ω*_4_	This work
BiOCl:3.0%Dy^3+^	7.51	1.83	3.49	*Ω*_2_ > *Ω*_6_ > *Ω*_4_	27
CaMoO_4_:3.0%Dy^3+^	7.78	0.96	2.92	*Ω*_2_ > *Ω*_6_ > *Ω*_4_	28
K_2_GdF_5_:Dy^3+^	2.51	0.94	2.12	*Ω*_2_ > *Ω*_6_ > *Ω*_4_	32
LiLuF_4_:Dy^3+^	2.04	0.91	1.09	*Ω*_2_ > *Ω*_6_ > *Ω*_4_	33
B_2_O_3_-BaO-Ga_2_O_3_:Dy^3+^	5.92	1.18	1.81	*Ω*_2_ > *Ω*_6_ > *Ω*_4_	34
NaBiSrP:0.1%Dy^3+^	2.94	0.14	1.65	*Ω*_2_ > *Ω*_6_ > *Ω*_4_	35
BaWO_4_:5.0%Dy^3+^	21.50	0.59	0.71	*Ω*_2_ > *Ω*_6_ > *Ω*_4_	36

The *Ω*_*λ*_ parameters contain important information about the crystal field; therefore, they can provide insights into the local environment surrounding the RE^3+^ ions. It is known that the *Ω*_*λ*_ parameters contain the following factor:^27,32,34^8
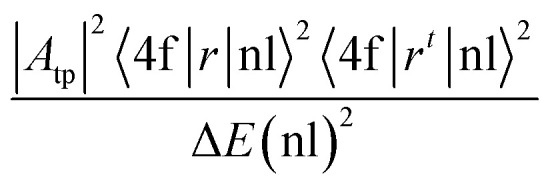
where the *A*_tp_ symbol represents the odd-order terms of the crystal field expansion. The *A*_tp_ parameters relate to the asymmetry of the crystal field. Δ*E*(nl) is the energy difference between the 4f^*n*^ and 4f^*n*−1^5d^1^ configurations. Δ*E*(nl) depends on the covalence of the RE^3+^-ligand bond. The remaining terms are the inter-configuration radial integrals with *t* = *λ* ± 1. For the *Ω*_2_ parameter, because *t* = 1 or 3, this parameter is less sensitive to the radial integrals, but is strongly influenced by the *A*_tp_ and Δ*E*(nl) quantities. The *Ω*_2_ magnitude of the CoAl_2_O_4_:Dy^3+^ NCs is higher than that of the materials such as K_2_GdF_5_:Dy^3+^,^32^ LiLuF_4_:Dy^3+^,^33^ and NaBiSrP: 0.1%Dy^3+^.^35^ This result indicates that the asymmetry of the ligand field in the CoAl_2_O_4_:Dy^3+^ NCs is higher than that in these materials. However, the *Ω*_2_ of CoAl_2_O_4_:Dy^3+^ NCs is smaller than that of some materials such as B_2_O_3_–BaO–Ga_2_O_3_:Dy^3+^,^34^ CaMoO_4_:Dy^3+^,^28^ BiOCl:Dy^3+^,^27^ and BaWO_4_:Dy^3+^.^36^ In this case, the low covalent degree of the Dy^3+^ ligand bond in the CoAl_2_O_4_ host could be the reason for the small value of the parameter *Ω*_2_. For *Ω*_6_, where *t* = 5 or 7, this parameter depends strongly on the radial integral 〈4f|*r*^*t*^|nl〉. A decrease in the covalent degree of the RE^3+^–ligand bond leads to an increase in the density of the 6s electrons, *i.e.*, an increase in the radial integral. Therefore, the high value of the *Ω*_6_ parameter in CoAl_2_O_4_:Dy^3+^ NCs suggests a low degree of covalency in the Dy^3+^–O^2−^ bond. The *Ω*_6_ parameter is influenced by the rigidity of the medium surrounding the RE^3+^ ions. As the vibration amplitude and average distance from the RE^3+^ ion to the nearest ligand cation increased, the *Ω*_6_ parameter also increased. Hence, a low value of the *Ω*_6_ parameter indicates high rigidity in the medium surrounding the RE^3+^ ions. Thus, a low value of the *Ω*_6_ parameter depicts a high rigidity of the medium surrounding the RE^3+^ ions. The *Ω*_6_ values (Table 2) in the CoAl_2_O_4_:Dy^3+^ NCs were larger than all *Ω*_6_ values in the other crystals. This result shows that the rigidity of the medium around the Dy^3+^ ion in the CoAl_2_O_4_ host was lower than that in the host materials.

Dy^3+^ is one of the two RE^3+^ ions commonly utilized as an optical probe to study the characteristics of the local environments surrounding RE^3+^ ions, with Eu^3+^ being the other ion. This application relies on the ligand-field dependence of the yellow/blue (Y/B) intensity ratio. To explain this property of the Dy^3+^ ion, some authors have argued that the ^4^F_9/2_ → ^6^H_15/2_ transition includes both the magnetic dipole (MD) and the electric dipole (ED); therefore, its intensity is less affected by the host.^32–35^ However, with Δ*J* = 3, this transition does not satisfy the selection rule of a magnetic dipole transition (Δ*J* = 0, ±1); thus, its MD probability is zero. In fact, the dependence of the Y/B ratio on the properties of the ligand field can be interpreted using JO theory. This theory shows that the probability of an ED transition is proportional to [*Ω*_2_‖*U*^(2)^‖^2^ + *Ω*_4_‖*U*^(4)^‖^2^ + *Ω*_6_‖*U*^(6)^‖^2^].^37^ Using the ‖*U*^(*λ*)^‖^2^ values in ref. 32, 34, and 35, Y/B is given by:9
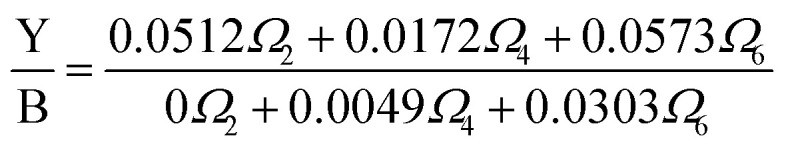
In JO theory, the yellow band strongly depends on *Ω*_2_, while the blue band is not affected by *Ω*_2_. Changes in the properties of the ligand field, such as asymmetry and polarizability, result in variations in the *Ω*_2_ parameter, which in turn significantly affects the Y/B intensity ratio. Therefore, in compounds doped with Dy^3+^ ions, the emission characteristics of the material can be tailored for specific applications by modifying the composition of the crystal host.

#### 3.4.2. Prediction of the fluorescence properties of CoAl_2_O_4_:Dy^3+^ NCs

Using the *Ω*_*λ*_ parameters, some properties of RE^3+^ ions such as the transition probabilities, branching ratios, and lifetime of any excitation level can be predicted. In this study, the radiative parameters of the transitions originating from the ^4^F_9/2_ and ^4^I_15/2_ levels as well as the lifetimes of these levels were computed. It has been reported that the probability of a transition from the J level to the J′ level is the sum of the MD and EM transition probabilities (*A*_MD_, and *A*_ED_):^38,39^

10 *A*_R_(J → J′) = *A*_ED_(J → J′) + *A*_MD_(J → J′)

The *A*_MD_ and *A*_ED_ values are given by the following expressions:^40^11
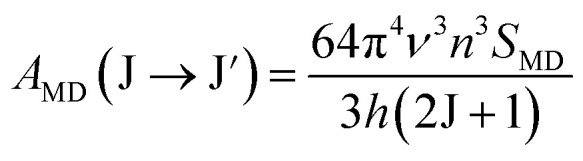
12
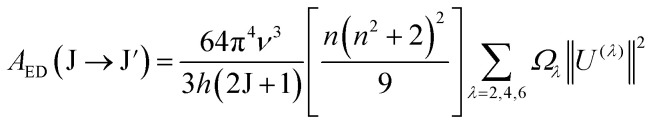
where *S*_MD_ denotes the strength of the magnetic dipole line. The *S*_MD_ only depends on the specific transition in RE^3+^ ions and is independent of the material and can be found in ref. 35, 36 and 40. The total transition probability from the J level to the lower J′ level is calculated using:^36,40^13
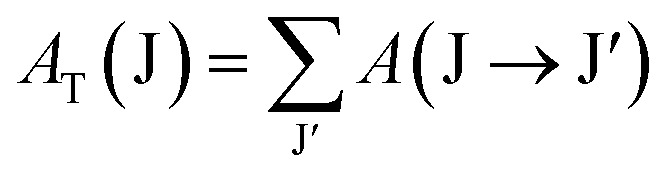


The branching ratio of a transition is calculated using the following expression:14
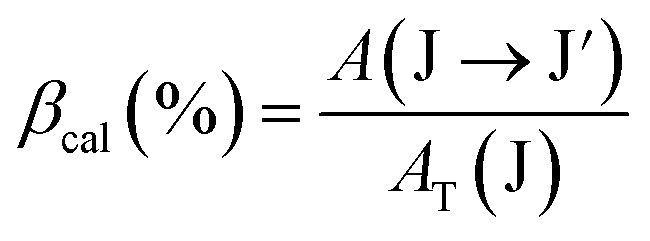


The lifetime (*τ*_cal_) of the J level is determined using the equation:15
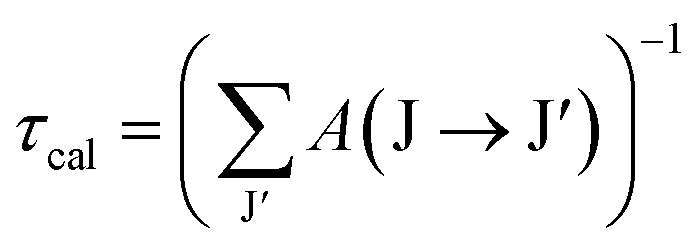


Using the above formulas, the radiative parameters of the ^4^F_9/2_ and ^4^I_15/2_ levels were predicted and are listed in Table 3. The spectral applications of Dy^3+^ ions are usually related to the ^4^F_9/2_ → ^6^H_15/2_ and ^4^F_9/2_ → ^6^H_13/2_ luminescence bands. For the CoAl_2_O_4_:Dy^3+^ NCs, the intensities of these bands were predicted to be dominant among the transitions originating from the ^4^F_9/2_ level. Their calculated branching ratios were 31.81 and 54.87%, respectively, whereas experimental results are 44.63 and 53.28%, respectively.

**Table 3:** Transition energies (*ν*), radiative parameters (*A*_MD_, *A*_ED_, and *A*_T_), and branching ratios (*β*) of the transitions originating from the ^4^F_9/2_ and ^4^I_15/2_ levels in the CoAl_2_O_4_:0.1%Dy^3+^ sample

Transition		*ν* (cm^−1^)	*A*_MD_ (s^−1^)	*A*_ED_ (s^−1^)	*A*_R_ (s^−1^)	*β*_cal_ (%)	*β*_exp_ (%)
^4^I_15/2_ → ^4^F_9/2_		1006	0	0.17	0.17	0.03	—
^4^I_15/2_ → ^6^F_1/2_		8498	0	0	0	0	—
^4^I_15/2_ → ^6^F_3/2_		9039	0	0.45	0.45	0.08	—
^4^I_15/2_ → ^6^F_5/2_		9807	0	0.21	0.21	0.04	—
^4^I_15/2_ → ^6^F_7/2_		11 203	0.36	0.36	0.72	0.07	—
^4^I_15/2_ → ^6^H_5/2_		12 002	0	0.32	0.32	0.06	—
^4^I_15/2_ → ^6^H_7/2_		13 049	0.25	1.66	1.91	0.31	—
^4^I_15/2_ → ^6^F_9/2_		13 137	0.23	16.49	16.72	3.06	—
^4^I_15/2_ → ^6^F_11/2_		14 485	0	33.06	33.06	6.13	—
^4^I_15/2_ → ^6^H_9/2_		14 511	3.23	8.03	11.26	1.49	—
^4^I_15/2_ → ^6^H_11/2_		16 344	0.89	19.08	19.97	3.55	—
^4^I_15/2_ → ^6^H_13/2_		18 668	0	61.46	61.46	11.39	—
^4^I_15/2_ → ^6^H_15/2_		22 148	0	397.96	397.96	73.79	—
***A***_**T**_ **(** ^**4**^ **I**_**15/2**_ **) = 539.27, *τ***_**cal**_ **(** ^**4**^ **I**_**15/2**_ **) = 1.854 ms**							
^4^F_9/2_ → ^6^F_1/2_	7492		0	0.08	0.08	0	—
^4^F_9/2_ → ^6^F_3/2_	8033		0	0.11	0.11	0.01	—
^4^F_9/2_ → ^6^F_5/2_	8801		0	4.46	4.46	0.49	—
^4^F_9/2_ → ^6^F_7/2_	10 197		0.43	4.97	5.4	0.59	—
^4^F_9/2_ → ^6^H_5/2_	10 996		0	3.65	3.65	0.39	—
^4^F_9/2_ → ^6^H_7/2_	12 043		0.32	18.07	18.39	2.01	54.87
^4^F_9/2_ → ^6^F_9/2_	12 131		0.29	7.14	7.43	0.81	31.81
^4^F_9/2_ → ^6^F_11/2_	13 479		0.06	17.02	17.08	1.86	—
^4^F_9/2_ → ^6^H_9/2_	13 505		4.20	14.43	18.63	2.03	—
^4^F_9/22_ → ^6^H_11/2_	15 338		1.180	45.76	46.94	5.12	2.09
^4^F_9/2_ → ^6^H_13/2_	17 662		0	553.01	503.01	54.87	53.28
^4^F_9/2_ → ^6^H_15/2_	21 142		0	241.58	291.58	31.81	44.63
***A***_**T**_ **(** ^**4**^ **F**_**9/2**_ **) = 916.76, *τ***_**cal**_ **(** ^**4**^ **F**_**9/2**_ **) = 1.091 ms**							

#### 3.4.3. Evaluation of the reliability of JO analysis

**Fig. 7:**
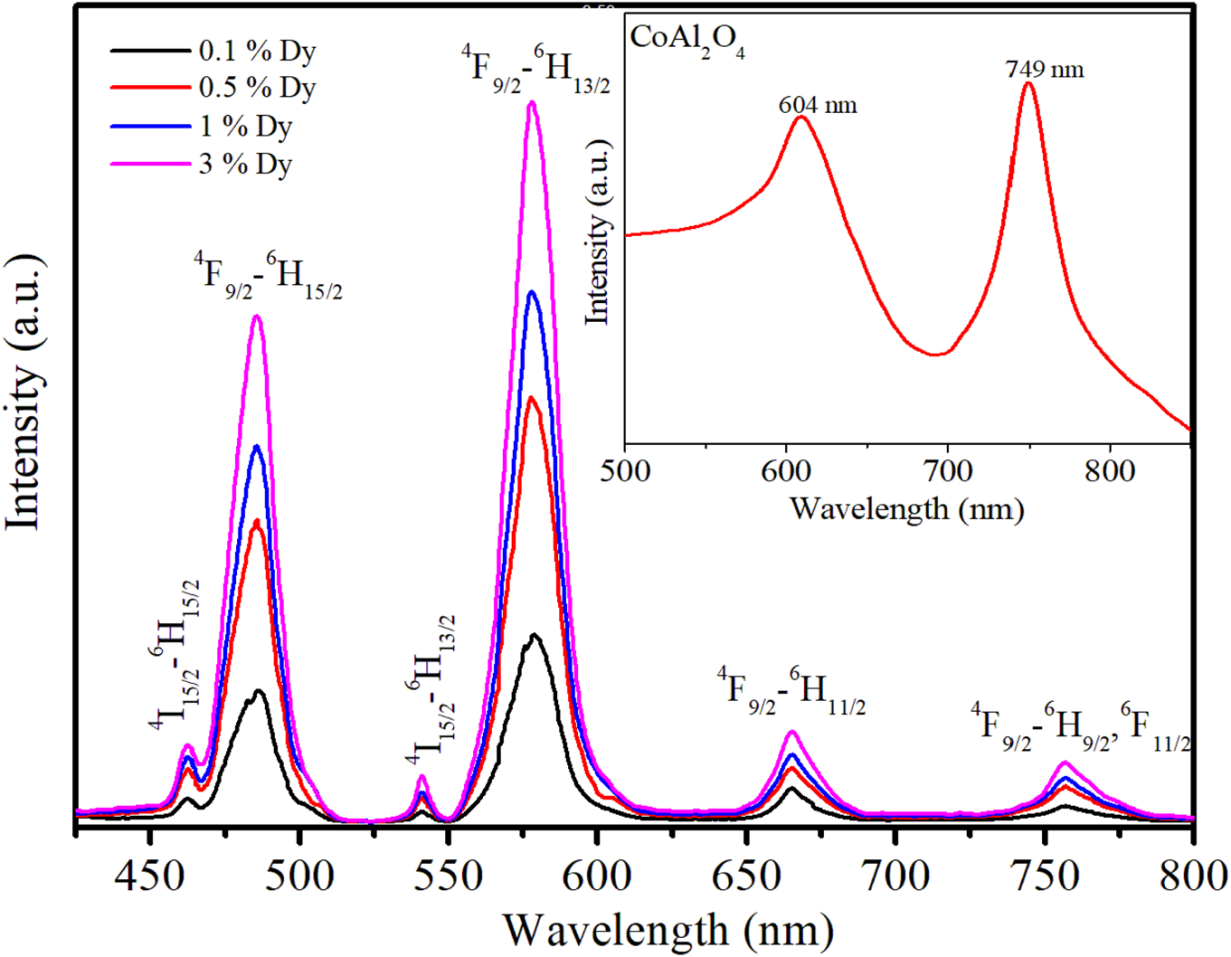
Emission spectra of the Dy^3+^-doped CoAl_2_O_4_NCs under excitation at 350 nm.

It is noted that the JO intensity parameters of Dy^3+^ ions are usually calculated from the absorption spectra. However, the *Ω*_*λ*_ parameters of the CoAl_2_O_4_:Dy^3+^ NCs in powder form were computed based on the luminescence excitation spectra. To verify the reliability of this calculation route, a three-level model was used. This model is based on thermalization between the closed levels. As shown in the emission spectra of the CoAl_2_O_4_:Dy^3+^ NCs, six emission peaks appear at 462, 482, 541, 579, 664, and 756 nm, corresponding to the transitions ^4^I_15/2_–^6^H_15/2_, ^4^F_9/2_–^6^H_15/2_, ^4^I_15/2_–^6^H_13/2_, ^4^F_9/2_–^6^H_13/2_, ^4^F_9/2_–^6^H_11/2_, and ^4^F_9/2_–^6^H_9/2_, ^6^F_11/2_ (see Fig. 7). After excitation by 350 nm UV irradiation, the Dy^3+^ ions are transferred to the ^6^P_7/2_ manifold and then relax rapidly to the ^4^F_9/2_ level through intermediate levels by the multi-phonon process. It is well known that the energy distance from the ^4^F_9/2_ level to the next lower level (^6^F_1/2_) is approximately 6500 cm^−1^, which is approximately 18 times the highest phonon energy in CoAl_2_O_4_. For such a large energy gap, the probability of the multi-phonon relaxation process can be ignored. Thus, the Dy^3+^ ions in the ^4^F_9/2_ level relax to the ground state through the emission process, which yields the characteristic luminescence bands of the Dy^3+^ ions.

The phonon energy of Dy^3+^ ions in CoAl_2_O_4_ NCs plays a crucial role in determining the optical and vibrational interactions within the material. Phonons represent the quantized vibrational energy of a crystal lattice, and when Dy^3+^ ions are doped in the CoAl_2_O_4_ host, they interact with these lattice vibrations. The energy of these phonons typically lies in the range of a few meV to several tens of meV, depending on the host material's lattice structure and bonding characteristics.^19^ This interaction can lead to non-radiative relaxation processes, where the excitation energy of the Dy^3+^ ions is transferred to the CoAl_2_O_4_ host lattice as heat, reducing luminescence efficiency. Additionally, the phonon energy can influence the splitting of energy levels within the Dy^3+^ ions, affecting their emission spectra and color purity.^40^ The crystal field of the CoAl_2_O_4_ host determines the local environment around the Dy^3+^ ions. It can further modulate the phonon energy, affecting the efficiency of optical transitions and the luminescence properties of Dy^3+^ ions.

In the Dy^3+^ ion, the energy distance between ^4^F_9/2_ and ^4^I_15/2_ levels is approximately several hundred cm^−1^.^19,40^ For this narrow energy gap, electrons can be transferred from the ^4^F_9/2_ level to the ^4^I_15/2_ level because of the thermal population even at room temperature. From the ^4^I_15/2_ level, Dy^3+^ ions may relax radiatively to the ground state. This process generates weak luminescence bands at wavelengths of approximately 462 and 541 nm corresponding to ^4^I_15/2_ → ^6^H_15/2_, and ^6^H_13/2_, respectively. In this case, the luminescence ratio from ^4^F_9/2_ and ^4^I_15/2_ levels can be described using a three-level model including ^6^H_15/2_ (level 0), ^4^F_9/2_ (level 1) and ^4^I_15/2_ (level 2):16
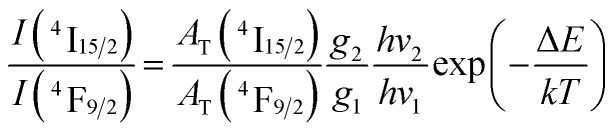
where *I*(^4^I_15/2_) and *I*(^4^F_9/2_) are the integrated intensities of the ^4^I_15/2_ → ^6^H_15/2_ and ^4^F_9/2_ → ^6^H_15/2_ luminescence bands, respectively; and *A*_T_(^4^I_15/2_) and *A*_T_(^4^F_9/2_) are the total emission probabilities of the ^4^I_15/2_ and ^4^F_9/2_ levels, respectively. These parameters were calculated using JO theory; *hν*_1_ and *hν*_2_ are the lowest and highest energies of the ^4^I_15/2_ → ^6^H_15/2_ and ^4^F_9/2_ → ^6^H_15/2_ bands, respectively; *g*_1_ and *g*_2_ are the degeneracies of the ^4^F_9/2_ and ^4^I_15/2_ states, respectively; Δ*E* is the energy distance from the highest stark level of the ^4^F_9/2_ state to the lowest stark level of the ^4^I_15/2_ states; *k* is the Boltzmann constant; and *T* = 300 K and *kT* = 201.6 cm^−1^. From the emission spectra of the CoAl_2_O_4_:0.1%Dy^3+^ sample, the *I*(^4^I_15/2_)/*I*(^4^F_9/2_) ratio is calculated to be 0.042. Through JO calculations, the *A*_T_(^4^I_15/2_) and *A*_T_(^4^F_9/2_) values were determined to be 539.27 and 916.76 s^−1^, respectively. Δ*E* was found to be 624 cm^−1^. This value is in good agreement with the energy separation from the highest stark level of the ^4^F_9/2_ state to the lowest stark level of ^4^I_15/2_ (∼564 cm^−1^), which was determined from the excitation spectra. The deviation between the calculation and experimental results was approximately 10%, which is within the allowable error range (20%) of JO analysis. This result shows that the calculation of the *Ω*_*λ*_ parameters based on the excitation spectra can be applied to Dy^3+^ ions doped into CoAl_2_O_4_ NCs.

#### 3.4.4. Radiative parameters of the ^4^F_9/2_ → ^6^H_13/2_ transition

From the emission spectra of the RE^3+^ ions, the experimental branching ratio (*β*_exp_) of the luminescence band was also determined. *β*_exp_ is the ratio between the integrated intensity of the luminescence band and the total emission intensity of all bands. For the CoAl_2_O_4_:Dy^3+^ NCs, the ^4^F_9/2_ → ^6^H_15/2_ and ^4^F_9/2_ → ^6^H_13/2_ transitions exhibited the highest intensities in the emission spectra. Therefore, some luminescence parameters (*e.g.* effective line width, stimulated emission cross-section, gain bandwidth, and optical gain) of the ^4^F_9/2_ → ^6^H_15/2_ and ^4^F_9/2_ → ^6^H_13/2_ transitions were calculated for all concentrations. The stimulated emission cross-section of an emission transition was calculated using the following formula:^36,40^17
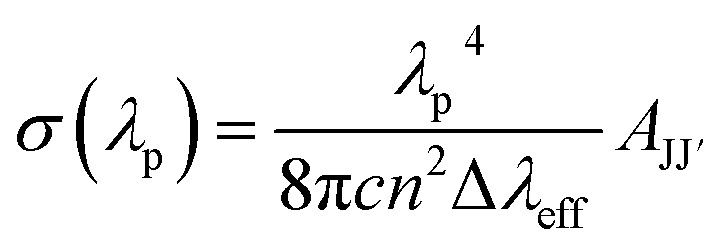
where Δ*λ*_eff_ denotes the effective line width of the emission band. The gain bandwidth and optical gain parameters were determined using the expressions (*σ*_*λ*_p__ × Δ*λ*_eff_) and (*σ*_*λ*_p__ × *τ*_R_), respectively. The calculated results are listed in Table 4. The large values of the stimulated emission cross-section, gain bandwidth and optical gain parameters of the yellow emission band suggest that the ^4^F_9/2_ → ^6^H_13/2_ transition in CoAl_2_O_4_:Dy^3+^ may be suitable for developing solid-state lasers as well as optical amplifier devices.

**Table 4:** The radiative parameters for the ^4^F_9/2_ → ^6^H_15/2_ and ^4^F_9/2_ → ^6^H_13/2_ transitions in CoAl_2_O_4_:*x*Dy^3+^ NCs: experimental branching ratio (*β*_exp_, %), effective bandwidth (Δ*λ*_eff_, nm), stimulated emission cross-section (*σ*_*λ*_p__, 10^−22^ cm^2^), gain bandwidth (*σ*_*λ*_p__ × Δ*λ*_eff_, 10^−28^ cm^3^), and optical gain (*σ*_*λ*_p__ × *τ*_R_, 10^−25^ cm^2^ s^−1^)

	*x* = 0.1%	*x* = 0.5%	*x* = 1.0%	*x* = 3.0%
^**4**^ **F**_**9/2**_ **→** ^**6**^ **H**_**15/2**_				
*β*_exp_	48.92	53.28	53.32	53.27
Δ*λ*_eff_	14.42	14.01	14.31	13.76
*σ*_*λ*_p__	5.18	5.61	5.92	5.91
*σ*_*λ*_p__ × Δ*λ*_eff_	7.46	78.60	84.72	81.32
*σ*_*λ*_p__ × *τ*_R_	5.88	6.12	5.96	6.32

^**4**^ **F**_**9/2**_ **→** ^**6**^ **H**_**13/2**_				
*β*_exp_	48.54	44.63	44.74	44.69
Δ*λ*_eff_	12.78	11.76	11.95	12.01
*σ*_*λ*_p__	27.76	31.32	33.38	30.49
*σ*_*λ*_p__ × Δ*λ*_eff_	35.48	36.83	39.89	36.62
*σ*_*λ*_p__ × *τ*_R_	31.51	34.17	33.61	32.62

### 3.5. Photoluminescence spectra and CIE color coordinates

**Fig. 8:**
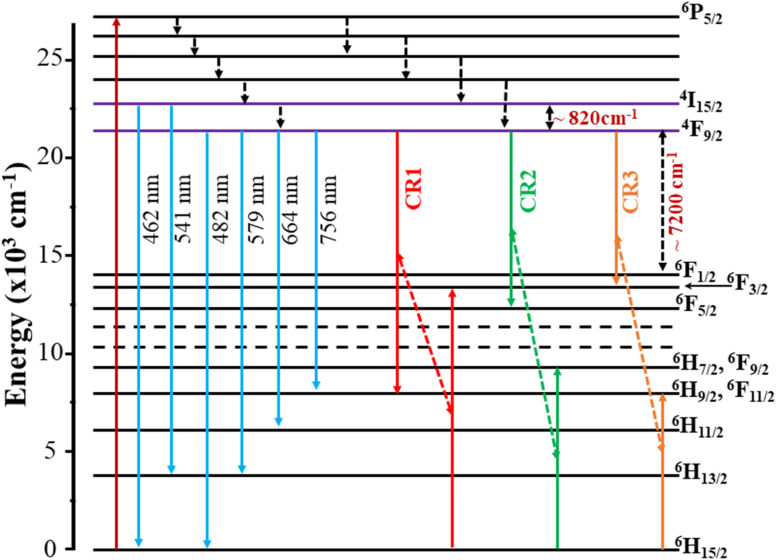
Energy level diagram and cross-relaxation channels of Dy^3+^ ions in CoAl_2_O_4_ NCs.

Fig. 7 shows the recorded emission spectra of the samples using an excitation wavelength of 350 nm from a xenon lamp source. The emission spectrum of the pure CoAl_2_O_4_ NCs (inset in Fig. 7) shows two emission peaks at 604 and 749 nm. These emission peaks originate from the electronic transitions within the Co^2+^ ions in the spinel structure. The emission peak at 604 nm was primarily due to the ^4^*T*_1_(P) → ^4^*A*_2_(F) transition of Co^2+^ ions in a tetrahedral coordination. This transition is spin-allowed and occurs within the d–d transitions of Co^2+^ ions.^8,19^ For Dy-doped CoAl_2_O_4_ NCs, six emission peaks appear at 462, 482, 541, 579, 664, and 756 nm, corresponding to the transitions ^4^I_15/2_–^6^H_15/2_, ^4^F_9/2_–^6^H_15/2_, ^4^I_15/2_–^6^H_13/2_, ^4^F_9/2_–^6^H_13/2_, ^4^F_9/2_–^6^H_11/2_, and ^4^F_9/2_–^6^H_9/2_, ^6^F_11/2_ (see Fig. 7 and 8). Among these bands, the yellow (Y) band at 579 nm, associated with the hypersensitive transition ^4^F_9/2_-^6^H_13/2_, and the blue (B) band at 482 nm, associated with the ^4^F_9/2_–^6^H_15/2_ transition, are the dominant transitions. Because of the well-understood properties of trivalent Dy^3+^ ions in various hosts, they are extensively used as spectroscopic probes. They provide valuable information regarding the structural and local symmetry of solid-state materials, making them useful in a wide range of applications in materials science and solid-state physics.^34,40^ As depicted in Fig. 7, the emission intensity of Dy^3+^ ions increased linearly with the concentration of Dy^3+^. This observation verifies that when the Dy concentration was up to 3%, there was no fluorescence quenching phenomenon in Dy-doped CoAl_2_O_4_ NCs.

The yellow luminescent peak (579 nm) predominantly arose from pure electric dipole transitions, making its intensity and shape highly dependent on changes in the environment surrounding the Dy^3+^ ion. Meanwhile, the blue luminescence peak (482 nm) was less sensitive to variations in the ligand field properties. Therefore, the ratio of the intensities of Y/B bands of the Dy^3+^ ion serves as a gauge for estimating the asymmetry of the ligand field. For the Dy-doped CoAl_2_O_4_ NCs, the Y/B ratio was always greater than 1 for all Dy^3+^ concentrations (Table 5). This observation suggests that the Dy^3+^ ions in Dy-doped CoAl_2_O_4_ NCs occupy asymmetric local environments, possibly because of the presence of an inversion center or other asymmetry-inducing factors.^41^

**Table 5:** The Y/B ratio, chromaticity coordinates (*x*, *y*) and correlated color temperature (CCT) of Dy-doped CoAl_2_O_4_ NCs

Sample	Y/B	*x*	*y*	CCT (K)
S01	1.45	0.3871	0.3956	3952.51
S05	1.42	0.3869	0.3975	3951.84
S1	1.41	0.3873	0.3973	3951.68
S3	1.43	0.3876	0.3999	3952.62

The McCamy empirical equation was used to estimate the CCT from the chromaticity coordinates. This equation is useful in the lighting field. The CCT is a specification of the color appearance of a light source and is derived from the color of light emitted by an ideal black-body radiator at a given temperature.

The CCT value can be evaluated using the McCamy equation:^42^

18 CCT = −449*n*^3^ + 3525*n*^2^ −6823*n* + 5520.33

**Fig. 9:**
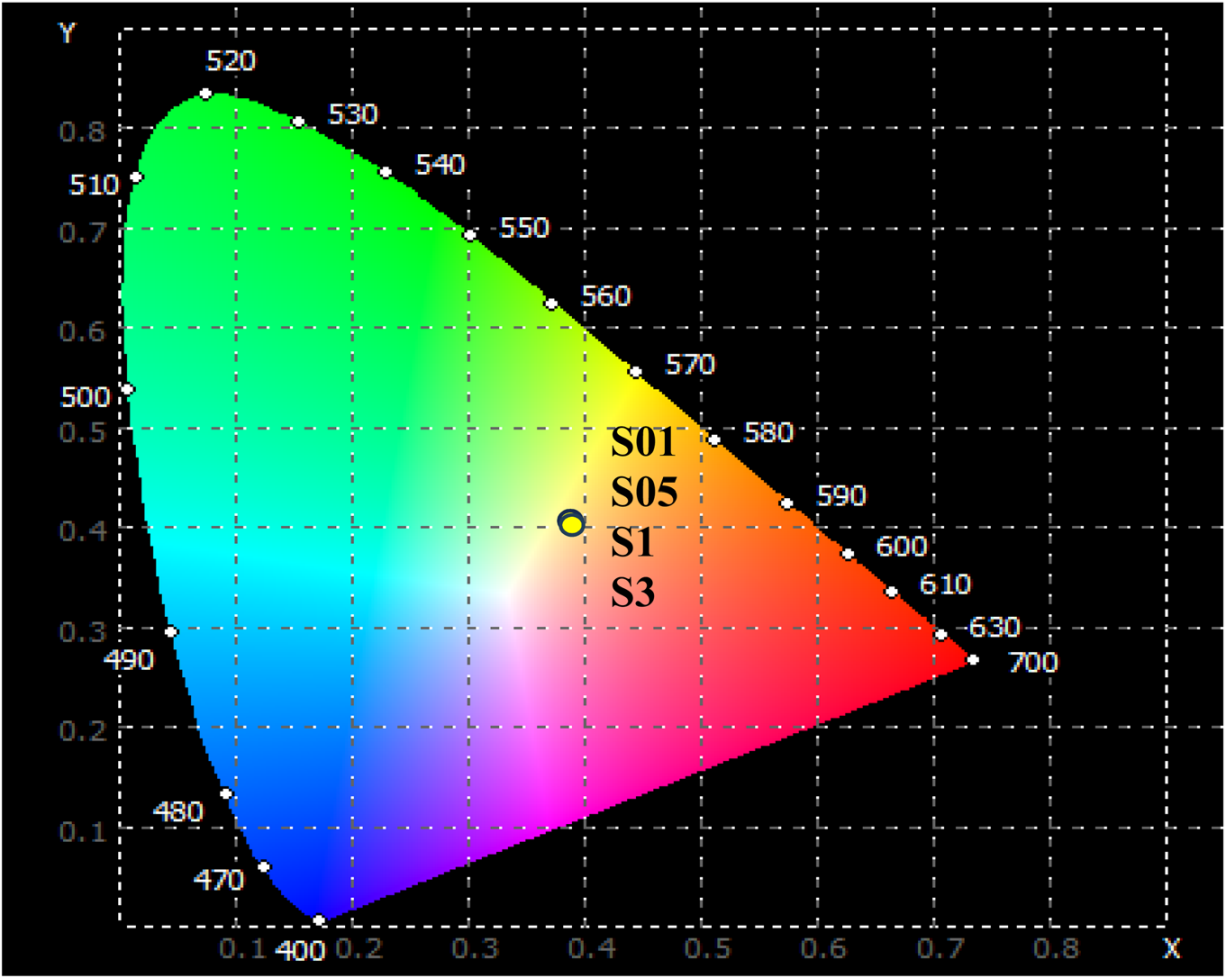
The CIE color coordinate diagram of CoAl_2_O_4_:Dy^3+^ NCs with *λ*_exc_ = 350 nm.

In the above formula, *x* and *y* are the chromaticity coordinates of the light source. *x*_e_ = 0.332, *y*_e_ = 0.186 are the chromaticity coordinates of the “epicenter”. *n* = (*x* − *x*_e_)/(*y* − *y*_e_) is the inverse-slope line. The CCT values of the Dy-doped CoAl_2_O_4_ NCs are presented in Table 5. The results showed that the CCT values depended very weakly on the concentration of Dy^3+^ ions (Fig. 9).

The CCT values were approximately 4000 K corresponding to a neutral or cool white color. CCT is common in office lighting, commercial spaces, and some residential areas where bright, white light is desired. These results indicate that the Dy-doped CoAl_2_O_4_ NCs show great potential for practical applications in displays and white LED devices when excited by UV radiation.

### 3.6. Luminescence decay curve analysis and energy transfer

**Fig. 10:**
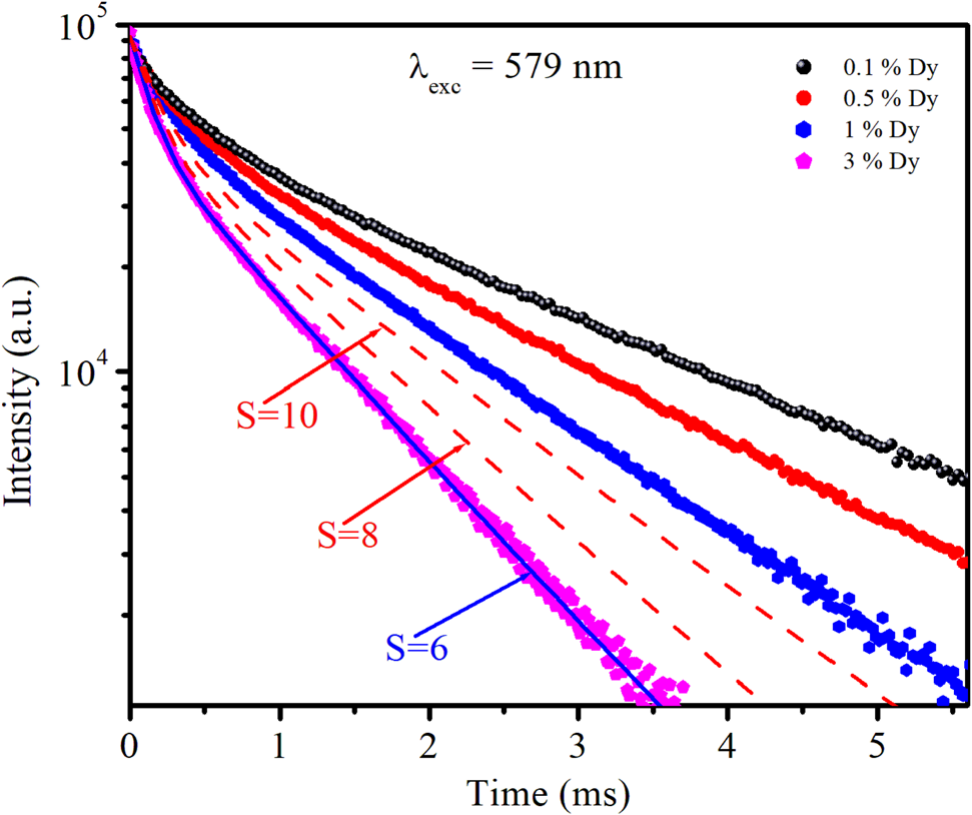
Decay time curves of CoAl_2_O_4_:Dy^3+^ NCs recorded at 579 nm (^4^F_9/2_ → ^6^H_13/2_), using an EPL-405 in the FLS1000.

The photoluminescence (PL) decay curves of the ^4^F_9/2_ → ^6^H_13/2_ transition (at 579 nm) for all samples are shown in Fig. 10. The PL decay curves are non-exponential because the emission process is influenced by multiple sources or energy transfer. The PL decay curves were fitted using a bi-exponential function:^19,43^19
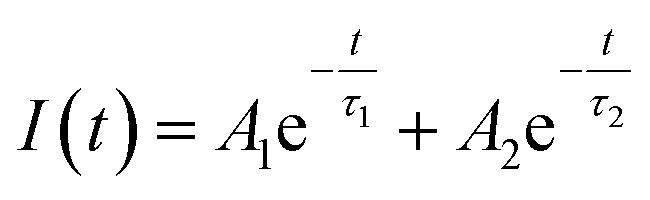
where *A*_*i*_ is the pre-exponential factor and *τ*_*i*_ is the lifetime.

The experimental lifetimes 〈*τ*〉 of samples were determined using the equation:^44^20
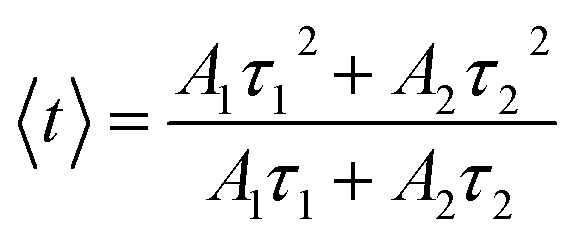
*A*_*i*_ and 〈*τ*〉 values obtained by fitting are listed in Table 6. The 〈*τ*〉 of the ^4^F_9/2_ level is found to be 1.03, 0.84, 0.69, and 0.36 ms for the S01, S05, S1, and S3 samples, respectively.

**Table 6:** Lifetime decay constants and fitness of curves

Sample	*τ*_1_ (ms)	*A*_1_	*τ*_2_ (ms)	*A*_2_	〈*τ*〉 (ms)	*R* ^2^
S01	0.66	0.365	1.16	0.635	1.03	0.9976
S05	0.56	0.312	0.92	0.688	0.84	0.9963
S1	0.42	0.384	0.78	0.616	0.69	0.9982
S3	0.26	0.342	0.4	0.658	0.36	0.9956

It can be observed that the lifetime decreases with increasing Dy concentration. Similar results were also observed for Dy-doped materials such as CdS,^45^ BaY_2_F_8_,^46^ and alumino-lithium-telluroborate.^41^ The lifetime decrease can be related to nonradiative recombination processes such as multiphonon relaxation and energy transfer (ET) between Dy^3+^ ions through cross-relaxation processes.^41^ However, multiphonon relaxation for Dy^3+^ ions cannot occur because the energy gap between the ^4^F_9/2_ and ^6^F_1/2_ states (approximately 7200 cm^−1^) is too large. Therefore, the decrease in the fluorescence lifetime with increasing Dy concentration is due to non-radiative energy transfer processes such as the cross-relaxation (CR) process between the donor and acceptor in Dy-doped CoAl_2_O_4_ NCs. In Dy^3+^ ions, CR channels play a significant role in nonradiative processes that affect photoluminescence properties. These channels involve energy transfer between ions and can be either resonant or nearly resonant: (i) CR1: (^4^F_9/2_ → ^6^H_9/2_) → (^6^H_15/2_ → ^6^F_3/2_), (ii) CR2: (^4^F_9/2_ → ^6^F_5/2_) → (^6^H_15/2_ → ^6^H_7/2_), and (iii) CR3: (^4^F_9/2_ → ^6^F_3/2_) → (^6^H_15/2_ → ^6^H_9/2_) (Fig. 8). These CR channels explain the complex interactions and energy transfer mechanisms in Dy^3+^-doped materials, which are critical for understanding their luminescence properties and potential applications in various optical devices.

The fluorescence quantum efficiency (*η*) is determined from the ratio between the experimental lifetime and the theoretically calculated lifetime and is given by the following equation:^19,41^21
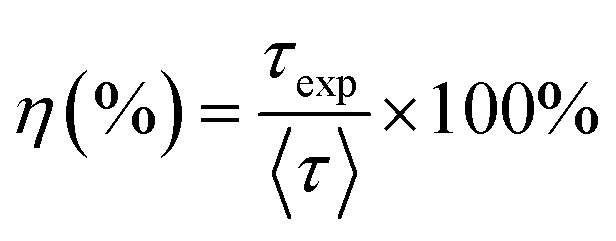


The ET rate (*W*_ET_) through CR was calculated using the following equation:^19,41^22
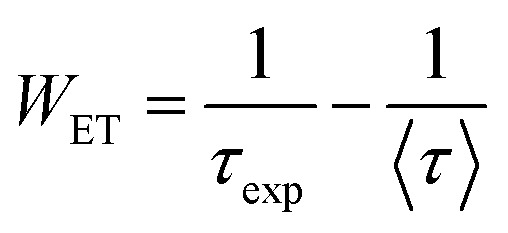


The *η* and *W*_ET_ values are calculated and given in Table 7. The results obtained in Table 7 show that as the Dy concentration increases, the quantum efficiency decreases while the energy transfer rate increases. The quantum efficiency decreases with increasing Dy ion concentration because the likelihood of nonradiative processes (such as cross-relaxation, energy migration to defects, and self-absorption) increases. These processes result in less energy being emitted as light and more being lost as heat. Conversely, the energy transfer rate increases because the closer proximity of ions enhances the probability and frequency of energy transfer interactions, leading to more efficient energy migration and cross-relaxation processes.

**Table 7:** Energy transfer parameters between Dy^3+^ ions in CoAl_2_O_4_:Dy^3+^ NCs

Sample	*τ*_R_ (ms)	〈*τ*〉 (ms)	*η* (%)	*W*_ET_ (s^−1^)	*R*_0_ (Å)	*Q*	*C*_DA_ (cm^6^ s^−1^)
S01	1.09	1.03	94.50	53.44	—	—	—
S05	0.93	0.84	90.32	115.21	7.21	0.72	1.51 × 10^−40^
S1	0.89	0.69	77.52	325.68	7.48	1.64	1.96 × 10^−40^
S3	0.71	0.36	50.70	1369.33	7.95	3.49	3.55 × 10^−40^

The Inokuti–Hirayama model is a powerful tool for analyzing luminescence decay curves owing to energy transfer processes among luminescent centers. This model helps understand the underlying mechanisms of energy transfer and provides quantitative parameters that describe these processes. The Inokuti–Hirayama model describes the decay of the photoluminescence intensity *I*(*t*) as a function of time *t*, incorporating both intrinsic luminescence decay and energy transfer to acceptor ions. The model is expressed by the formula:^19,37,47^23
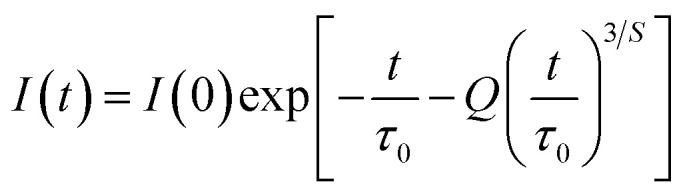
where *I*(0) is the initial luminescence intensity and *τ*_0_ is the intrinsic lifetime of the luminescent center in the absence of energy transfer. For CoAl_2_O_4_:Dy^3+^ NCs, *τ*_0_ is regarded as the lifetime of the ^4^F_9/2_ level at a Dy^3+^ ion concentration of 0.1 mol% because energy transfer can be disregarded at this concentration. *S* is a parameter that depends on the nature of the multipolar interactions between ions: *S* = 6 for dipole–dipole (D–D) interaction, 8 for dipole-quadrupole (D–Q) interaction, and 10 for quadrupole–quadrupole (Q–Q) interaction; *Q* is a parameter that depends on the concentration of acceptor ions, defined as:^37,41^24
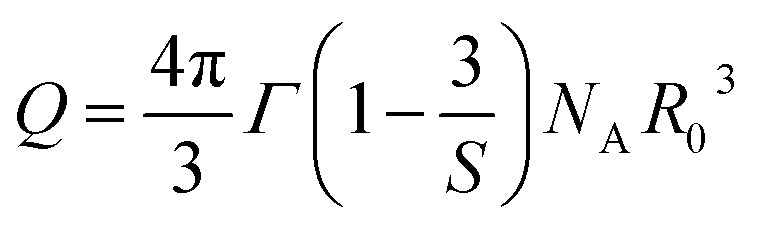
where *N*_A_ is the concentration of acceptor ions, which is considered as the concentration of Dy^3+^ ions, *R*_0_ is the critical distance between the Dy^3+^ ions at which the radiative probability of the donor is equal to the ET probability from the donor to acceptors, and *Γ* is Euler's gamma function (*Γ* = 1.77 for D–D interaction, *Γ* = 1.43 for D–Q interaction, and *Γ* = 1.3 for Q–Q interaction). The fitted the decay curves of the ^4^F_9/2_ level to eqn (23) are shown in Fig. 10. The decay curves are best fitted with *S* = 6 (the red lines in Fig. 10), indicating that D–D interaction is the dominant mechanism in the energy transfer process between Dy^3+^ ions in CoAl_2_O_4_:Dy^3+^ NCs. Dominant D–D interaction between Dy^3+^ ions has also been observed in various hosts, such as semiconductor CdS,^45^ BaY_2_F_8_,^46^ zinc fluorophosphate,^48^ alumino-lithium-telluroborate,^41^ and bismuthate glasses.^49^ The critical transfer distance (*R*_0_) was calculated according to the ET parameter (*Q*) and the concentration of Dy^3+^. The interaction constant between Dy^3+^ ions was calculated using the equation *C*_DA_ = *R*_0_^6^·*τ*_0_^−1^. *τ*_0_ is the lifetime of the ^4^F_9/2_ level in the CoAl_2_O_4_:0,1%Dy^3+^ sample (*τ*_0_ = 1.03 ms). The calculated results are listed in Table 7. The results in Table 7 show that the values of *Q*, *R*_0_, and *C*_DA_ increased with increasing Dy^3+^ ion concentration. The calculated results show that the Dy^3+^ ion concentration strongly affects the parameters *Q* and *C*_DA_.

## 4 Conclusion

The co-precipitation method was used to successfully synthesize CoAl_2_O_4_:Dy^3+^ NCs. The prepared NCs formed a spinel structure with a particle size of approximately 24 nm. The color characteristics of Dy^3+^ luminescence in the CoAl_2_O_4_ NCs were evaluated using CIE chromaticity coordinates and correlated color temperatures. The PLE spectra were used to calculate the optical parameters of the Dy^3+^ ions in the CoAl_2_O_4_ host using Judd–Ofelt theory. In this analysis, the *Ω*_*λ*_ parameters were calculated using the PLE spectra, and the reliability of the calculations was verified using a three-level model. The Inokuti–Hirayama model was used to study the energy transfer process between the Dy^3+^ ions. The dipole–dipole interaction is the dominant mechanism in the energy transfer process between Dy^3+^ ions in CoAl_2_O_4_:Dy^3+^ NCs. The decrease in the fluorescence lifetime of the ^4^F_9/2_ level with increasing Dy concentration is related to the energy transfer process between Dy^3+^ ions through the cross-relaxation channels: (^4^F_9/2_ → ^6^H_9/2_) → (^6^H_15/2_ → ^6^F_3/2_), (^4^F_9/2_ → ^6^F_5/2_) → (^6^H_15/2_ → ^6^H_7/2_), and (^4^F_9/2_ → ^6^F_3/2_) → (^6^H_15/2_ → ^6^H_9/2_). The large values of the stimulated emission cross-section, gain bandwidth and optical gain parameters of the yellow emission band suggest that the ^4^F_9/2_ → ^6^H_13/2_ transition in CoAl_2_O_4_:Dy^3+^ NCs is suitable for developing solid-state lasers as well as optical amplifier devices.

## Conflicts of interest

There are no conflicts to declare.

## Data Availability

The data supporting this study's findings are available on request from the corresponding author [Nguyen Xuan Ca, email: nguyenxuanca@tnus.edu.vn]. The data are not publicly available due to privacy reasons.

## References

[cit1] Feng C., Yin W. J., Nie W., Zu X., Huda M. N., Wei S. H., Al-Jassim M. M., Turner J. A., Yan Y. (2012). Appl. Phys. Lett..

[cit2] Xuanmeng H., Wang F., Liu H., Niu L., Wang X. (2018). J. Am. Ceram. Soc..

[cit3] Gao H., Yang H., Wang S., Danming Li., Wang F., Fang L., Lei L., Xiao Y., Yang G. (2018). J. Sol-Gel Sci. Technol..

[cit4] Taguchi M., Nakane T., Hashi K., Ohki S., Shimizu T., Sakka Y., Matsushita A., Abe H., Funazukuria T., Nakab T. (2013). Dalton Trans..

[cit5] Pradhan S. K., Dalal B., Sarkar A., De S. K. (2019). Phys. Chem. Chem. Phys..

[cit6] Wang Y., Wang S., Yu X., Tang S., Han S., Yang L. (2020). Optik.

[cit7] Tong Y., Zhang H., Wang S., Chen Z., Bian B. (2016). J. Nanomater..

[cit8] Tang Y., Wu C., Song Y., Zheng Y., Zhao K. (2018). Ceram. Int..

[cit9] Irshad A., Shahid M., Bahy S. M. E., Azab I. H. E., Mersal G. A. M., Ibrahim M. M., Agboola P. O., Shakir I. (2022). Phys. B.

[cit10] Yang L., Mu Z., Zhang S., Wang Q., Zhu D., Zhao Y., Luo D., Zhang Q., Wu F. (2018). J. Mater. Sci.: Mater. Electron..

[cit11] Wang Q., Mu Z., Yang L., Zhang S., Zhu D., Yang Y., Luo D., Wu F. (2018). Phys. B.

[cit12] Haritha P., Martín I. R., Viswanath C. S. D., Vijaya N., Krishnaiah K. V., Jayasankar C. K., Haranath D., Lavín V., Venkatramu V. (2017). Opt. Mater..

[cit13] Kumar K. U., Silva W. F., Krishnaiah K. V., Jayasankar C. K., Jacinto C. (2014). J. Nanophotonics.

[cit14] Sołtys M., Gorny A., Pisarska J., Pisarski W. A. (2018). Opt. Mater..

[cit15] Dharmaiah P., Viswanath C. S. D., Basavapoornima C., Krishnaiah K. V., Jayasankar C. K., Hong S. J. (2016). Mater. Res. Bull..

[cit16] Li X. Y., Shen L. F., Pun E. Y. B., Lin H. (2023). Ceram. Int..

[cit17] Sahu I. P. (2016). J. Mater. Sci.: Mater. Electron..

[cit18] Shamsi A., Hashemian S. (2020). Desalin. Water Treat..

[cit19] Kien N. T., Lam V. D., Duong P.
V., Hien N. T., Luyen N. T., Do P. V., Binh N. T., Ca N. X. (2024). RSC Adv..

[cit20] Yu H., Chen S., Chen J. (2017). IOP Conf. Ser.: Mater. Sci. Eng..

[cit21] Duan X., Pan M., Yu F., Yuan D. (2011). J. Alloys Compd..

[cit22] Peng X., Cheng J., Yuan J., Jin N., Kang J., Hou Y., Zhang Q. (2018). Adv. Appl. Ceram..

[cit23] Bamford C. R. (1962). Phys. Chem. Glasses.

[cit24] Radhika S. P., Sreeram K. J., Nair B. U. (2012). J. Adv. Ceram..

[cit25] Zhang Y., Chen B., Xu S., Li X., Zhang J., Sun J., Zhang X., Xia H., Hua R. (2018). Phys. Chem. Chem. Phys..

[cit26] Luo M., Chen B., Li X., Zhang J., Xu S., Zhang X., Cao Y., Sun J., Zhang Y., Wang X., Zhang Y., Gao D., Wang L. (2020). Phys. Chem. Chem. Phys..

[cit27] Shivakumara C., Saraf R. (2016). Dyes Pigm..

[cit28] Dutta S., Som S., Sharma S. K. (2015). RSC Adv..

[cit29] Boudiafa S., Nasrallaha N., Mellalb M., Belabedc C., Belhamdid B., Mezianie D., Mehdic B., Trari M. (2020). Optik.

[cit30] Luo W., Liao J., Lia R., Chen X. (2010). Phys. Chem. Chem. Phys..

[cit31] Carnal W. T., Fields P. R., Rajnak K. (1968). J. Chem. Phys..

[cit32] Do P. V., Tuyen V. P., Quang V. X., Thanh N. T., Thai Ha V. T., Tuyen H. V., Khaidukov N. M., Marcazzó J., Lee Y. I., Huy B. T. (2013). Opt. Mater..

[cit33] Bigotta S., Tonelli M., Cavalli E., Belletti A. (2010). J. Lumin..

[cit34] Pisarski W. A. (2022). Materials.

[cit35] George H., Deopa N., Kaur S., Prasad A., Sreenivasulu M., JAyasimhadri M., Rao A. S. (2019). J. Lumin..

[cit36] Kunti A. K., Patra N., Sharma S. K., Swart H. C. (2018). J. Alloys Compd..

[cit37] Do P. V., Ca N. X., Thanh L. D., Quan D. D., Hung N. M., Du P. T., Huong N. T., Anh D. T. (2023). Phys. Chem. Chem. Phys..

[cit38] Rajesh D., Ratnakaramn Y. C., Seshadri M., Balakrishna A., Krishna T. S. (2012). J. Lumin..

[cit39] Xiong H. H., Shen L. F., Pun E. Y. B., Lin H. (2014). J. Lumin..

[cit40] Lakshminarayanaa G., Baki S. O., Lira A., Caldiño U., Meza-Rocha A. N., Kityk I. V., Abas A. F., Alresheedi M. T., Mahdi M. A. (2018). J. Non-Cryst. Solids.

[cit41] Phi Tuyen V., Xuan Quang V., Van Do P., Duy Thanh L., Xuan Ca N., Xuan Hoa V., Van Tuat L., Anh Thi L., Nogami M. (2019). J. Lumin..

[cit42] McCamy C. S. (1992). Color Res. Appl..

[cit43] Cuong K. C., Thuy N. T. M., Fan X., Hao P. V., Quynh L. K., Huong T. T. T., Kien N. T., Van N. T. K., Hien N. T., Dung L. N., Ca N. X. (2023). Chem. Phys. Lett..

[cit44] Ca N. X., Hien N. T., Do P. V., Yen V. H., Cuong K. C., Thu P. N., Lam L. T., Dung L. N., Quynh L. K., Hao P. V. (2023). RSC Adv..

[cit45] Ca N. X., Do P. V., Thuy N. T. M., Binh N. T., Hien N. T., Tan P. M., Hien B. T. T., Chi T. T. K. (2023). Opt. Mater..

[cit46] Parisi D., Toncelli A., Tonelli M., Cavalli E., Bovero E., Belletti A. (2005). J. Phys.: Condens.Matter.

[cit47] Lu J., Mu Z., Zhu D., Wang Q., Wu F. (2018). J. Lumin..

[cit48] Vijaya N., Upendra K., Jayasankar C. K. (2013). Spectrochim. Acta, Part A.

[cit49] Pisarskia W. A., Pisarska J., Lisiecki R., Dzik G. D., Romanowski W. R. (2012). Chem. Phys. Lett..

